# Cardiac Remote Monitoring Devices and Technologies: A Review for the Perioperative Physician and Telemedicine Providers

**DOI:** 10.7759/cureus.53914

**Published:** 2024-02-09

**Authors:** Rohini Kotha, Caleb Streitmatter, Andrew Serdiuk, Nasrin N Aldawoodi, Robert S Ackerman

**Affiliations:** 1 Anesthesiology, H. Lee Moffitt Cancer Center & Research Institute, Tampa, USA; 2 Medicine, University of South Florida Morsani College of Medicine, Tampa, USA

**Keywords:** cardiac remote monitoring devices, telehealth, telemedicine in pre-anesthesia testing, telehealth for the perioperative area, remote patient monitoring in preoperative assessment, remote patient monitoring devices, remote patient monitoring, remote cardiac monitoring

## Abstract

Cardiovascular complications are a major cause of morbidity and mortality after surgery, necessitating adequate and thorough preoperative risk stratification and screening. Several technological advances in cardiac remote monitoring have improved the assessment and diagnosis of cardiovascular disease in patients before and after surgery. These devices perform measurements of physiological function, including vital signs, and more advanced functions, such as electrocardiograms and heart sound recordings. Some of the currently available devices include Fitbit® (Google LLC, Mountain View, CA, USA), BodyGuardian® (Preventive Inc., Rochester, MN, USA), Zephyr^TM^ Performance Systems (Zephyr Inc., Annapolis, MD, USA), Sensium® (The Surgical Company, Amersfoort, UT, The Netherlands), KardiaMobile® (AliveCor, Mountain View, CA, USA), Coala® Heart Monitor (Coala Life Inc., Uppsala, Sweden), Smartex® Wearable Wellness System (Smartex, Porto, LX, Portugal), Eko® CORE and DUO (Eko Health, Emeryville, CA, USA), and TytoCare^TM ^(TytoCare Ltd., New York, USA). Early studies have applied these devices to asymptomatic individuals and those with known cardiovascular disease with good sensitivity and specificity for electrophysiologic diagnosis. These devices carry several technical and other limitations, somewhat restricting the generalization of their use to all patients. However, information gathered from these devices can further guide anesthetic technique, operative timing, and postoperative follow-up, among other variables. As telehealth becomes more prevalent and comprehensive, it is paramount for the perioperative physician to be familiar with the available cardiac remote monitoring technologies.

## Introduction and background

Cardiac complications are the leading cause of morbidity and mortality after non-cardiac surgery, with myocardial infarction (MI) reported in as many as 3% of patients undergoing major surgery [1]. Formulating safe preoperative assessments requires an understanding of the patient’s overall status, including previously diagnosed cardiovascular disease (CVD) or the identification of risk factors for CVD. Key to the preoperative cardiac risk assessment is the decision of whom to evaluate, the calculation and categorization of the risk of major adverse cardiac events (MACE), and the estimation of functional capacity [2].

Per the 2014 American College of Cardiology (ACC) and American Heart Association (AHA) guidelines on perioperative cardiovascular evaluation and management of patients undergoing non-cardiac surgery, patients with known CVD, elevated (>1%) risk of MACE based on a validated risk calculator, or less than four metabolic equivalents (MET) are often sent for further cardiac evaluation [3, 4]. The average resting adult oxygen consumption is approximately 3.6 mL/kg/min, analogous to one MET. The ability to complete four METs has been primarily deemed an indication of suitability for surgery [5]. Risk factors, as described by the Revised Cardiac Risk Index, include a history of ischemic heart disease, congestive heart failure, or cerebrovascular disease, preoperative insulin treatment, preoperative creatinine > 2 mg/dL, and high-risk surgery status [6]. In the spectrum of MI risk stratification, detection, and diagnosis, many components of the patient’s cardiac status must be ascertained, including cardiopulmonary fitness, physiological reserve, and troponin screening [1].

Another regular decision of the perioperative physician is to determine if preoperative cardiac testing is indicated. A review of the ACC/AHA 2014 guidelines suggests an ECG for patients with known CVD and a likelihood of CVD in symptomatic individuals [3]. Furthermore, an echocardiogram is indicated for those with a history of heart failure and clinical status change, unexplained dyspnea, or undergoing high-risk surgery. Stress testing is considered for patients undergoing high-risk surgery with low or unknown functional capacity [3].

To further add to the perioperative physician’s evaluation of patients with risk factors for CVD, remote cardiac monitors have been utilized to obtain further information regarding a patient’s baseline vital signs, physiologic status, and cardiac rhythms and dysrhythmias. Furthermore, they can be incorporated into telemedicine techniques to expand on the virtual physical examination. This review introduces and highlights existing technologies to remotely monitor patients while emphasizing appropriate indications and limitations (summarized in Table 1).

**Table 1:** A summary of the various remote cardiac monitoring devices available on the market and their notable functionality, indications, and limitations. AF: Atrial fibrillation

Device name	Functions	Proposed benefits
Sensium® Patch (The Surgical Company, Amersfoort, UT, The Netherlands)	Single lead ECG, respiratory rate, and temperature	Inpatient monitoring in high-risk patients pre- and post-op
Fitbit® (Google LLC, Mountain View, CA, USA)	Single lead ECG with arrhythmia detection, physical activity, and sleep patterns	Prognostic tool in stable ischemic heart disease and post-op patients
BodyGuardian® HEART (Preventive Inc., Rochester, MN, USA)	ECG with arrhythmia detection, respiratory rate, and activity monitoring	QT prolongation monitoring and screening for permanent pacemaker implantation
Zephyr^TM^ Performance Systems (Zephyr Inc., Annapolis, MD, USA)	Blood pressure, heart rate, heart rate variability, pulse oximetry, and weight measurement	Long-term monitoring in heart failure and prognostic tool in pre- and post-op monitoring; potential reduction in healthcare utilization in remote populations
Coala® Heart Monitor (Coala Life Inc., Uppsala, Sweden)	Two lead ECG and synchronized heart sound recording with automatic arrhythmia detection	Post-stroke AF monitoring and remote reduction in cardiac palpitation-induced anxiety
Smartex® Wearable Wellness System (Smartex, Porto, LX, Portugal)	Two lead ECG, respiratory rate, and triaxial accelerometer integrated into the vest	Early in testing; developed for use in populations suffering from chronic lung disease
KardiaMobile® (AliveCor, Mountain View, CA, USA)	Single lead ECG for arrhythmia detection and QRS elongation	Remote screening during isolated cardiac symptoms; potential diagnostic tool
Eko® CORE and DUO (Eko Health, Emeryville, CA, USA)	Cardiac auscultation with AF and murmur detection	Telehealth cardiac auscultation for pediatric and adult patients
TytoCare^TM ^(TytoCare Ltd., New York, USA)	Examination camera, speaker, infrared thermometer, digital stethoscope, digital otoscope, and tongue depressor	Telehealth tool for clinician-guided caregivers to carry out noninvasive medical examinations

## Review

Fitbit®

One remote monitoring device (RMD) with well-tested heart rate (HR) recording capabilities and good user satisfaction is Fitbit from Google [6]. A study from the Journal of the AHA collected biometric and psychometric data from patients with stable ischemic heart disease and compared this to levels of biomarkers for MACE risk. This analysis revealed a high correlation between daily physical activity and lower serum levels of MACE biomarkers, indicating a potential to predict decreases in these biomarkers based on daily physiologic data [7].

Another study utilized Fitbit to monitor patients pre- and post-op for pancreatic surgery. A Fitbit and smartphone were used to record HR, activity patterns, sleep, and screen time with daily symptom surveys. This study then created models that could predict if the following day would be a "high symptom" day, with 73.5% accuracy based on Fitbit and smartphone data [8].

In addition to symptom prediction, arrhythmia detection has also been studied extensively. A remote clinical trial enrolled 450,000 patients to examine the validity of a novel photoplethysmography (PPG)-based software algorithm for detecting atrial fibrillation (AF) with a fitness tracker. Upon detecting an irregular heart rhythm, participants received a single-lead ECG patch monitor for one week along with the fitness tracker [9]. A total of 1000 individuals with irregular rhythms underwent subsequent ECG monitoring, and AF was detected in 32%. The novel algorithm had a positive predictive value of 98.2% for detecting AF in patients with undiagnosed AF [10]. While these studies do not suggest that Fitbit is a high-accuracy diagnostic tool, they demonstrate that it can assess and predict patient outcomes in many clinical scenarios.

BodyGuardian®

One notable device, the BodyGuardian HEART by Preventice Inc., consists of an adhesive patch with skin electrodes and a sensor module for data collection, recording, and transmission of HR and respiratory rate (RR) biometric data. A small pilot study clinically validated the BodyGuardian system's HR and RR monitoring accuracy by comparing it to traditional in-clinic measurements. A strong correlation was found between the HR measurements of the traditional and the BodyGuardian wearable device. However, the RR showed a poor correlation [11].

Another pilot study conducted in 2016 tested the BodyGuardian system on its ability to record quality electrocardiographs and detect harmful rhythms such as AF in 10 healthy patients of an assisted living center. Four ECG technicians determined the quality of the BodyGuardian ECGs to be of minimum diagnostic quality or better with clear QRS complexes for 88% of the recordings. Based on the heart rhythm classification of the ECG technicians, the BodyGuardian system had a 97% sensitivity and a 77% specificity for determining AF [12].

Castelletti et al. also compared the BodyGuardian system with a 12-lead Holter monitor to assess how accurately the former could identify QT interval prolongation on an ECG for early identification of torsades de pointes. The BodyGuardian system was used to continuously record ECG and automatically measure the QT interval of healthy and long QT syndrome patients. Of the total 351 measurements, only 13 were found to be incorrect, and the impact of the disagreement between the BodyGuardian system and the Holter monitor only resulted in the misclassification of one patient out of 36 [13].

Zephyr^TM^ Performance Systems

The Zephyr BioHarness device wirelessly relays HR, RR, heart rate variability (HRV), and accelerometry data to a computer to be analyzed by an algorithm. The Zephyr BioHarness chest strap utilizes contact electrodes and capacitive sensors to determine HR and RR, respectively [14]. A validation study from 2013 assessed the ability of the Zephyr BioHarness chest strap to accurately determine HR and RR during two exercise protocols compared to a 12-lead ECG. The Spearman correlation coefficients of the two systems' measurements were highly correlated, with a range of R-values from 0.87 to 0.96 for the HR measurements and 0.80 to 0.97 for the RR measurements [15].

Heart rate variability is valuable as a measure of physiological state, especially in athletes, as it can estimate a person's autonomic nervous system activity [16]. Stress has been shown to decrease HRV, which has been linked to poor emotion regulation and easy mental exhaustion [17-19]. The predictive capacity of HRV has significant potential in pre- and post-surgical monitoring. One systematic review analyzed 10 studies completed between 2010 and 2017 utilizing the Zephyr BioHarness [20]. The HRV of the BioHarness was determined to be very reliable, with a standard error of measurement value ranging from 2.11 to 5.90 bpm and a test/re-test reliability coefficient of ≥0.85. In three studies, the HR biases were less than 3.00 bpm, with 95% limits of agreement of -3.10 to 2.42. In addition to the contact electrode system of the BioHarness, PPG has been shown to provide a reliable measurement of HRV.

The Zephyr BioHarness system has shown substantial accuracy in measuring HR and HRV and can be a potentially helpful tool for remote monitoring. However, most of the studies cited were performed on young and healthy individuals during exercise. Further study is needed to evaluate its benefit in a clinical setting.

Sensium® Patch

The Sensium Patch from The Surgical Company is another lightweight wearable biosensor worn on the patient's chest that monitors HR using single-lead ECG, RR through impedance pneumography, and axillary temperature using a temperature-sensitive resistor. It can detect abnormalities and notify healthcare staff immediately when measurements reach a threshold. The Sensium link software can be installed on the hospital network, allowing patient data to be seamlessly conveyed to clinical staff [21].

A randomized controlled trial compared remote monitoring after major elective abdominal surgery with Sensium to standard monitoring with the National Early Warning Score (NEWS) system alone. Downey et al. used data from the Trail of Remote Continuous vs. Intermittent NEWS Monitoring (TRaCINg) trial. They determined that patients were less likely to have an unplanned critical care admission and had shorter average lengths of hospital stay in the Sensium plus NEWS arm compared to intermittent NEWS monitoring alone [22]. There was no difference in the time taken to receive antibiotics in the setting of sepsis, and Sensium had the potential to be cost-saving compared to standard NEWS monitoring alone. Most patients found the patch comfortable and felt safe wearing it [22].

Hernandez-Silveira et al. performed a study to assess the correlation between Sensium and a widely used vital signs monitor, the Philips IntelliVue MP30 (Philips Medical Systems, Best, NB, The Netherlands) [23]. Sensium provided reliable information on HR for patients with normal sinus rhythm, and valid HR data was reported most of the time (typically >80%), even in the presence of abnormal QRS morphology. For RR, valid data was reported 50% of the time. However, the collected data had good accuracy, with a mean difference of less than one breath per minute compared to the bedside monitor. Most of the RR data for patients with AF was rejected as invalid, similar to the HR data [23]. Sensium's RR measurement accuracy was also tested in a prospective observational study of adult patients acutely admitted to a medical ward, with staff and standardized clinical measurements as a reference. The agreement was inadequate in both comparisons, suggesting that the accuracy of Sensium's impedance pneumography may be too low for daily clinical practice applications [24].

Sensium was used in a small proof-of-concept remote-COVID trial of 10 individuals with mild suspected COVID-19 symptoms requiring quarantine. The Sensium patch was applied on arrival for the duration of their stay, and alerts were generated when pre-established thresholds were reached. Ten vital alerts were generated across participants, resulting in telephone contact, reassurance, or sensor adjustment [25]. No one required hospitalization or a virtual general practitioner review, and both participants and healthcare staff reported favorable experiences. Continuous monitoring with Sensium may facilitate predictive modeling for deterioration and early interventions in remote and community settings [25].

KardiaMobile®

The cardiac remote monitoring devices currently available can perform basic physiologic and rhythm monitoring with ECG collection and analysis. Many consumer products, like smartwatches, are integrating ECG functions into their capabilities, although these tend to record less detailed and accurate readings than their medical counterparts. One remote monitoring device that shows promise is the KardiaMobile ECG monitor. This is a 1-lead ECG system that uses a small finger pad connected to a smartphone to detect AF, bradycardia, tachycardia, normal sinus rhythm, premature ventricular contractions, supraventricular ectopy, and wide QRS [26]. A clinical validation study utilized the KardiaMobile device for patients presenting with palpitations at an outpatient cardiology clinic [27]. A total of 33 patients were monitored for 14 to 30 days with a KardiaMobile device and a LifeWatch (LifeWatch Technologies Ltd., Tel Aviv-Jaffa, Israel) external loop recorder (ELR). The recorded ECGs from the two devices were read by two blinded cardiologists. Compliance with the ELR device was poor compared to the KardiaMobile device. Of the 33 patients, 100% had arrhythmias recorded by the KM device, while the ELR found arrhythmias in only 72% of these patients. The KardiaMobile device found symptomatic arrhythmias in 34% of the recorded days, while the ELR only found them in 20%. These results show that, compared to the ELR, the KardiaMobile device found a potential diagnosis in more patients and had greater adherence due to its ease of use [28].

The KardiaMobile system was also used in a more extensive study assessing patients presenting with paroxysmal AF, palpitations, or near collapse at a private cardiology clinic [29]. Patients were instructed to use the KardiaMobile system whenever they experienced symptoms. The ECGs were interpreted by the KardiaMobile algorithm and by a team consisting of a cardiologist, a cardiology nurse, and a doctor's assistant. The cardiology team confirmed AF in 80% of the ECGs that KardiaMobile categorized as AF, with most remaining ECGs confirmed as sinus rhythm. From these results, this study found the KardiaMobile device to have a sensitivity of 92% and a specificity of 95% for diagnosing AF [29].

A similar study compared the KardiaMobile device to a standard 12-lead monitor for 99 patients in an inpatient setting [30]. The ECGs were recorded with the KardiaMobile device and simultaneously with a 12-lead monitor as a gold standard. The cardiologists determined that 80% of the KardiaMobile ECGs were of adequate diagnostic quality. The sensitivity and specificity of detecting AF with a cardiologist interpreting the KardiaMobile ECGs were 100% and 94%, respectively. In comparison, the KardiMobile algorithm had a sensitivity and specificity for detecting AF of 70% and 69%, respectively.

Coala Heart Monitor®

Another device, the Coala Heart Monitor, came to the market in 2016 and has since been attracting attention as an effective and easy-to-use remote monitoring tool [31]. This device captures a 2-lead ECG synchronized with heart sound recordings. Based on the manual interpretation of 1000 ECGs, the sensitivity and specificity of the Coala algorithm were determined to be 95% and 98%, respectively [32].

One study monitored 100 stroke patients with the Coala device to determine the device's efficacy in detecting AF missed in the initial evaluation [33]. After the initial stroke evaluation with routine testing, patients used the Coala device for 28 days, recording ECGs twice a day and during symptoms. Nine patients (9%) had ECGs indicating the diagnosis of AF, with the first episode of AF occurring at a mean of 12.7 +/- 7.8 days. This same study assessed patient experience and overall satisfaction on a visual analog scale, with a median score correlating to a "very favorable" opinion of the device [34].

The use of the monitor to immediately confirm the benign status of cardiac symptoms has also been shown to improve health-related quality of life and reduce anxiety-related symptoms [35-37]. While this device requires some level of physical and cognitive effort, which may be impaired in stroke patients, these studies show the Coala Heart Monitor to have both a clinical benefit and a high degree of patient satisfaction.

Smartex® Wearable Wellness System

The goal of advancing remote ECG monitoring technology is to provide cost-effective, high-quality, and safe patient care that is convenient and palatable to the healthcare consumer. One of the earliest practical applications of remote monitoring was published by Sweeney et al. [38]. The investigators combined ECG, respiration, and acceleration sensors to identify sleep apneic events. Obstructive sleep apnea (OSA) is a prevalent yet treatable cause of significant morbidity and mortality. The researchers theorized that a screening tool for OSA before a sleep study could significantly reduce healthcare costs. This study used the Smartex Wearable Wellness System, which incorporates a two-lead ECG, a knitted textile stretch receptor embedded in the front of the system to measure respiratory excursion, and a triaxial accelerometer to measure body position. Five patients had their Smartex data analyzed post-polysomnography (PSG) testing. Of the three modalities, the combination of ECG and accelerometer had the highest accuracy, and the authors suggest that this tool could be used to monitor treatment response [38].

Eko® CORE and DUO

Eko is an FDA-approved ECG-enabled digital and electronic stethoscope. It is one of the first digital stethoscopes to integrate artificial intelligence (AI) diagnostic support. There are two different products with this diagnostic support service: the Eko CORE and the Eko DUO.

Eko CORE is a cylindrical digital attachment that can be inserted between the distal tubing and diaphragm of a standard stethoscope. It can amplify heart, lung, and body sounds and wirelessly pair to a free Health Insurance Portability and Accountability Act (HIPAA)-compliant Eko mobile app to visualize, record, share, and annotate heart sound waveform/phonocardiogram (PCG). The Eko DUO is a small, rectangular device that can be attached to earpieces or used wirelessly when paired with a smartphone. In addition to heart sounds and PCG, the Eko DUO can add a single-lead EKG.

Researchers from the Mayo Clinic used the Mayo Clinic Digital Data Vault to train a convolutional neural network AI model to identify patients with ventricular dysfunction (defined as ejection fraction ≤35%) and AF using AI-enhanced ECG data [39,40]. This AI model has been studied as part of the Phono- and Electrocardiogram-Assisted Detection of Valvular Disease (PEA-Valve) study for aortic stenosis and mitral regurgitation, where patients undergoing transthoracic echocardiograms also underwent PCG recordings by the Eko CORE device [41,42].

Eko's deep learning algorithm is comparable to that of expert cardiologists and pediatric cardiologists in its ability to detect heart murmurs, clinically significant aortic stenosis, and mitral regurgitation [42,43]. The PCG using Eko CORE can be a valuable tool to enhance the physical examination of patients with left ventricular assist devices (VADs). Changes in sound profile may reflect underlying device pathology [44]. Acoustic signals from VADs can allow early detection of pump thrombosis, the most common cause of failure in VADs, before triggering clinical events [45].

One of the greatest advantages of Eko is the tele-auscultation of pediatric and adult patients. The data files generated from Eko can be electronically shared via Bluetooth through a smartphone app and downloaded for storage and sharing. There is also an option to live-stream the examination in real time. A physician can direct the entire exam when combined with standard telemedicine applications involving two-way audio and video.

Simply put, Eko CORE has a high percentage of agreement with in-person auscultatory and echocardiographic findings with moderate inter-rater reliability. There are certain qualitative differences between the recorded sounds and in-person auscultation, but users were able to acclimate to these subtle differences. The system was considered easy to use, and most cardiologists in the study would consider using it in certain clinical settings [46]. In a Canadian systematic review and utilization study of digital stethoscopes for cardiopulmonary assessments, the Eko DUO, combined with the app, was ranked the best in audio quality and ease of use [47].

TytoCare^TM^

The TytoCare device is an FDA-approved comprehensive medical examination solution for remote physical examination of the heart, lungs, ears, throat, skin, abdomen, and temperature. TytoCare offers a modular medical device with an exam camera, thermometer, and adaptors that function as an otoscope, stethoscope, and tongue depressor [48].

A prospective randomized study was performed at an outpatient pediatric cardiology clinic to assess the clinical validity and reliability of the TytoCare device and compare it with the One Digital Stethoscope (Thinklabs, Centennial, CO, USA) and Horus HD Digital Scope System (Jedmed, St. Louis, MO, USA). The images and sounds were collected by a nurse and randomized by the device prior to review by eight physicians blinded to the device and subject. The quality of images and sounds obtained using TytoCare was judged to be higher than those obtained using stand-alone remote examination devices. It was shown to enable remote diagnosis and enable clinicians to assess abnormal clinical findings more adequately and correctly. There was a high level of intra- and inter-rater reliability for the recorded measurements of the heart, lungs, and ears [49].

Discussion

As a result of the preoperative cardiac risk assessment, testing, and evaluation, changes to anesthetic technique and agent, as well as intraoperative management, may be made, including the selection of volatile or intravenous maintenance anesthetics, neuraxial or regional anesthesia modalities, intraoperative transesophageal echocardiography, hemodynamic assist device use, arterial and central access and monitoring, and pulmonary artery catheterization, among others [50]. Transesophageal echocardiography provides real-time information regarding hemodynamics, great vessel pathology, and cardiac valve function [51]. The focused cardiac point-of-care ultrasound can also assess cardiac preload, afterload, and myocardial contractility and provide further diagnostic evaluation for possible hypovolemia, aortic stenosis, valvular disease, and pulmonary embolism [52].

Cardiac output non-invasive monitors have been recently employed to further intraoperative assessments of fluid responsiveness and tissue perfusion [53]. The majority of perioperative ischemic events are transient, preceded by a heart rate increase, and can often be treated with beta-blockade. Continuous online ECG monitoring has yet to significantly reduce postoperative cardiac complications via early detection and prevention of prolonged ischemia [54].

The TeleHealth Ten is a patient-assisted physical examination to guide virtual encounters beyond history-taking and interviewing. The 10-step checklist includes a neck assessment for jugular venous distension, possible wheezing or tachypnea, and peripheral pitting edema via thumb pressure, among other vital signs [55]. Risk factors for morbidity and mortality postoperatively can be potentially modified if identified and treated adequately before surgery. Prehabilitation interventions can include respiratory muscle training, sleep hygiene, aerobic conditioning, lifestyle modification, and others [56].

Telemedicine can improve the delivery of care to patients by reducing costs while achieving excellent outcomes in various fields, including anesthesiology [57]. A randomized pilot trial of telemedicine and in-person pre-anesthesia evaluations demonstrated equivalency in procedure cancellations, difficult airway predictions, and heart and lung physical examinations while significantly satisfying patients and providers [58]. A retrospective analysis further supports these findings, suggesting improvements in time spent, distance traveled, and overall financial burden without an increase in day-of-surgery cancellations or postponements [59]. Remote vital sign monitoring, telecommunications, and advanced technological devices can extend expert care to more patients with increased efficiency [60].

## Conclusions

Telemedicine was initially developed to provide basic care to underserved patients. Since the coronavirus pandemic, higher rates of telemedicine use have been seen nationally. Telehealth can improve the delivery of care to patients by reducing costs while achieving higher patient satisfaction and better outcomes. Patients and clinicians have enjoyed the benefits of telehealth, but limitations in performing physical examinations and monitoring can be a barrier to widespread use. Remote vital sign monitoring, telecommunications, and advanced technological devices can extend expert care to more patients with increased efficiency. From the anesthesiology perspective, this can include remote preoperative evaluation, expert collaboration, consultation for intraoperative and postoperative management, furthering educational opportunities, and communication effectiveness. This review shares the knowledge and successes of remote monitoring devices to improve a technology-driven perioperative arena.

## References

[1] Sellers D, Srinivas C, Djaiani G (2018). Cardiovascular complications after non-cardiac surgery. Anaesthesia.

[2] Smilowitz NR, Berger JS (2020). Perioperative cardiovascular risk assessment and management for noncardiac surgery: a review. JAMA.

[3] Raslau D, Bierle DM, Stephenson CR, Mikhail MA, Kebede EB, Mauck KF (2020). Preoperative cardiac risk assessment. Mayo Clin Proc.

[4] Fleisher LA, Fleischmann KE, Auerbach AD (2014). 2014 ACC/AHA guideline on perioperative cardiovascular evaluation and management of patients undergoing noncardiac surgery: a report of the American College of Cardiology/American Heart Association Task Force on Practice Guidelines. Circulation.

[5] Douglas N, Andrews G, Altamimi H, Wang A, Basto J, Smith RE, Taylor HE (2022). Real-world estimate of the value of one metabolic equivalent in a population of patients planning major surgery. Intern Med J.

[6] (2022). Fitbit Official Site for Activity Trackers & More. https://www.fitbit.com/global/us/home.

[7] Shufelt CL, Kim A, Joung S (2020). Biometric and psychometric remote monitoring and cardiovascular risk biomarkers in ischemic heart disease. J Am Heart Assoc.

[8] Low CA, Li M, Vega J (2021). Digital biomarkers of symptom burden self-reported by perioperative patients undergoing pancreatic surgery: prospective longitudinal study. JMIR Cancer.

[9] Lubitz SA, Faranesh AZ, Atlas SJ (2021). Rationale and design of a large population study to validate software for the assessment of atrial fibrillation from data acquired by a consumer tracker or smartwatch: the Fitbit Heart Study. Am Heart J.

[10] (2022). Novel algorithm on wearable devices can detect irregular heartbeat, may prompt early care | American Heart Association. https://newsroom.heart.org/news/novel-algorithm-on-wearable-devices-can-detect-irregular-heartbeat-may-prompt-early-care?preview=a26fc8680231ad282984a55ddb5e91c6..

[11] Izmailova ES, McLean IL, Hather G (2019). Continuous monitoring using a wearable device detects activity-induced heart rate changes after administration of amphetamine. Clin Transl Sci.

[12] Bruce CJ, Ladewig DJ, Somers VK (2016). Remote electrocardiograph monitoring using a novel adhesive strip sensor: a pilot study. World J Cardiol.

[13] Castelletti S, Dagradi F, Goulene K, Danza AI, Baldi E, Stramba-Badiale M, Schwartz PJ (2018). A wearable remote monitoring system for the identification of subjects with a prolonged QT interval or at risk for drug-induced long QT syndrome. Int J Cardiol.

[14] (2022). Wearable patient monitor - BioHarness™ 3 - Zephyr - compact / ECG / intensive care. https://www.medicalexpo.com/prod/zephyr/product-83995-684688.html.

[15] Kim JH, Roberge R, Powell JB, Shafer AB, Jon Williams W (2013). Measurement accuracy of heart rate and respiratory rate during graded exercise and sustained exercise in the heat using the Zephyr BioHarness. Int J Sports Med.

[16] Task Force of the European Society of Cardiology the North American Society of Pacing Electrophysiology (1996). Heart rate variability — standards of measurement, physiological interpretation, and clinical use. Circulation.

[17] Park G, Thayer JF (2014). From the heart to the mind: cardiac vagal tone modulates top-down and bottom-up visual perception and attention to emotional stimuli. Front Psychol.

[18] Park G, Van Bavel JJ, Vasey MW, Thayer JF (2012). Cardiac vagal tone predicts inhibited attention to fearful faces. Emotion.

[19] Williams DP, Cash C, Rankin C, Bernardi A, Koenig J, Thayer JF (2015). Resting heart rate variability predicts self-reported difficulties in emotion regulation: a focus on different facets of emotion regulation. Front Psychol.

[20] Nazari G, Bobos P, MacDermid JC, Sinden KE, Richardson J, Tang A (2018). Psychometric properties of the Zephyr BioHarness device: a systematic review. BMC Sports Sci Med Rehabil.

[21] (2022). Sensium Healthcare TZ202020R1 Wireless Patient Monitoring Device - SensiumBridge User Manual TZ202000 IFU. https://usermanual.wiki/Sensium-Healthcare/TZ202020R1/pdf.

[22] Downey CL, Croft J, Ainsworth G (2020). Trial of remote continuous versus intermittent NEWS monitoring after major surgery (TRaCINg): a feasibility randomised controlled trial. Pilot Feasibility Stud.

[23] Hernandez-Silveira M, Ahmed K, Ang SS (2015). Assessment of the feasibility of an ultra-low power, wireless digital patch for the continuous ambulatory monitoring of vital signs. BMJ Open.

[24] Granholm A, Pedersen NE, Lippert A, Petersen LF, Rasmussen LS (2016). Respiratory rates measured by a standardised clinical approach, ward staff, and a wireless device. Acta Anaesthesiol Scand.

[25] Iqbal FM, Joshi M, Davies G, Khan S, Ashrafian H, Darzi A (2021). The pilot, proof of concept REMOTE-COVID trial: remote monitoring use in suspected cases of COVID-19 (SARS-CoV 2). BMC Public Health.

[26] (2022). Arrhythmia Detections | Kardia. https://kardia.com/detections.

[27] Narasimha D, Hanna N, Beck H (2018). Validation of a smartphone-based event recorder for arrhythmia detection. Pacing Clin Electrophysiol.

[28] Sana F, Isselbacher EM, Singh JP, Heist EK, Pathik B, Armoundas AA (2020). Wearable devices for ambulatory cardiac monitoring: JACC state-of-the-art review. J Am Coll Cardiol.

[29] Selder JL, Breukel L, Blok S, van Rossum AC, Tulevski II, Allaart CP (2019). A mobile one-lead ECG device incorporated in a symptom-driven remote arrhythmia monitoring program. The first 5,982 Hartwacht ECGs. Neth Heart J.

[30] Wegner FK, Kochhäuser S, Ellermann C (2020). Prospective blinded evaluation of the smartphone-based AliveCor Kardia ECG monitor for atrial fibrillation detection: the Peak-AF study. Eur J Intern Med.

[31] (2022). About us - Coala Life Inc.. https://www.coalalife.com/us/about/..

[32] Olson A (2018). Symptom-ruled real-world arrhythmic recordings with an Internet-based system. Circulation.

[33] Magnusson P, Lyren A, Mattsson G (2020). Diagnostic yield of chest and thumb ECG after cryptogenic stroke, Transient ECG Assessment in Stroke Evaluation (TEASE): an observational trial. BMJ Open.

[34] Magnusson P, Lyren A, Mattsson G (2020). Patient-reported feasibility of chest and thumb ECG after cryptogenic stroke in Sweden: an observational study. BMJ Open.

[35] Insulander P, Carnlöf C, Schenck-Gustafsson K, Jensen-Urstad M (2020). Device profile of the Coala Heart Monitor for remote monitoring of the heart rhythm: overview of its efficacy. Expert Rev Med Devices.

[36] Carnlöf C, Insulander P, Jensen-Urstad M (2019). Instant analysis of the ECG with a new digital technique during palpitations reduce symptoms, anxiety, depression, and increase HRQOL in women. Eur Heart J.

[37] Insulander P, Carnlöf C, Jensen-Urstad M (2019). Symptomatic palpitations causing anxiety in women, what are the underlying arrhythmias. Euro Heart J.

[38] Sweeney K, Mitchell E, Gaughran J (2013). Identification of sleep apnea events using discrete wavelet transform of respiration, ECG and accelerometer signals. IEEE Int Conf Body Sens Netw.

[39] Attia ZI, Kapa S, Lopez-Jimenez F (2019). Screening for cardiac contractile dysfunction using an artificial intelligence-enabled electrocardiogram. Nat Med.

[40] Attia ZI, Noseworthy PA, Lopez-Jimenez F (2019). An artificial intelligence-enabled ECG algorithm for the identification of patients with atrial fibrillation during sinus rhythm: a retrospective analysis of outcome prediction. Lancet.

[41] Roy TS, Roy JK, Mandal N (2023). Design and development of electronic stethoscope for early screening of valvular heart disease prediction. Biomed Signal Process Control.

[42] Chorba JS, Shapiro AM, Le L (2021). Deep learning algorithm for automated cardiac murmur detection via a digital stethoscope platform. J Am Heart Assoc.

[43] Maidens J, Slamon NB (2018). Artificial intelligence detects pediatric heart murmurs with cardiologistlevel accuracy. Circulation.

[44] Araj F, Amin A, Cox J (2019). Refining auscultation of left ventricular assist devices: insights from phonocardiography. VAD J.

[45] Semiz B, Hersek S, Pouyan MB (2020). Detecting suspected pump thrombosis in left ventricular assist devices via acoustic analysis. IEEE J Biomed Health Inform.

[46] Behere S, Baffa JM, Penfil S, Slamon N (2019). Real-world evaluation of the Eko electronic Teleauscultation system. Pediatr Cardiol.

[47] Koning C, Lock A (2021). A systematic review and utilization study of digital stethoscopes for cardiopulmonary assessments. J Med Res Innov.

[48] (2022). TytoCare. https://www.tytocare.com/.

[49] McDaniel NL, Novicoff W, Gunnell B, Cattell Gordon D (2019). Comparison of a novel handheld telehealth device with stand-alone examination tools in a clinic setting. Telemed J E Health.

[50] Patel AY, Eagle KA, Vaishnava P (2015). Cardiac risk of noncardiac surgery. J Am Coll Cardiol.

[51] Fox J, Glas K, Swaminathan M, Shernan S (2005). The impact of intraoperative echocardiography on clinical outcomes following adult cardiac surgery. Semin Cardiothorac Vasc Anesth.

[52] Haskins SC, Tanaka CY, Boublik J, Wu CL, Sloth E (2017). Focused cardiac ultrasound for the regional anesthesiologist and pain specialist. Reg Anesth Pain Med.

[53] Funk DJ, Moretti EW, Gan TJ (2009). Minimally invasive cardiac output monitoring in the perioperative setting. Anesth Analg.

[54] Landesberg G (2005). Monitoring for myocardial ischemia. Best Pract Res Clin Anaesthesiol.

[55] Benziger CP, Huffman MD, Sweis RN, Stone NJ (2021). The Telehealth Ten: A guide for a patient-assisted virtual physical examination. Am J Med.

[56] McCann M, Stamp N, Ngui A, Litton E (2019). Cardiac prehabilitation. J Cardiothorac Vasc Anesth.

[57] Galvez JA, Rehman MA (2011). Telemedicine in anesthesia: an update. Curr Opin Anaesthesiol.

[58] Applegate RL II, Gildea B, Patchin R (2013). Telemedicine pre-anesthesia evaluation: a randomized pilot trial. Telemed J E Health.

[59] Aldawoodi NN, Muncey AR, Serdiuk AA, Miller MD, Hanna MM, Laborde JM, Getting REG (2021). A retrospective analysis of patients undergoing telemedicine evaluation in the preanesthesia testing clinic at H. Lee Moffitt Cancer Center. Cancer Control.

[60] Bridges KH, McSwain JR (2021). Telemedicine for anesthesiologists. Anesthesiol Clin.

